# Intrapatient tumour heterogeneity and clonal evolution in an autopsy study of metastatic salivary gland cancer

**DOI:** 10.1002/1878-0261.70315

**Published:** 2026-08-06

**Authors:** Gerben Lassche, Niels J. van Ruitenbeek, Charlotte Adang, Luke O'Gorman, Pui Yuen Lee, Willemijn M. Klein, Adriana C. H. van Engen‐van Grunsven, Jack A. Schalken, Sander Bervoets, Gerald W. Verhaegh, Carla M. L. van Herpen

**Affiliations:** ^1^ Department of Medical Oncology, Radboud Institute for Medical Innovation Radboud University Medical Center Nijmegen The Netherlands; ^2^ Radboud Technology Center for Bioinformatics, Radboud Institute for Molecular Life Sciences Radboud University Medical Center Nijmegen The Netherlands; ^3^ Department of Pathology, Radboud Institute for Medical Innovation Radboud University Medical Center Nijmegen The Netherlands; ^4^ Department of Medical Imaging, Radboud Institute for Medical Innovation Radboud University Medical Center Nijmegen The Netherlands; ^5^ Department of Urology, Radboud Institute for Medical Innovation Radboud University Medical Center Nijmegen The Netherlands

**Keywords:** adenoid cystic carcinoma, autopsy, clonal evolution, myoepithelial carcinoma, salivary gland cancer, tumour heterogeneity

## Abstract

Genetic tumour heterogeneity, driven by clonal evolution, contributes to therapy resistance in many cancers. Most salivary gland cancers, including adenoid cystic carcinoma (AdCC) and myoepithelial carcinoma (MECA), lack effective systemic treatments, highlighting the need to characterise their evolutionary profiles. In this autopsy study, we reconstructed genetic heterogeneity and clonal evolution in two patients with metastatic AdCC and one patient with metastatic MECA. Radiology‐guided autopsy was performed between 12 and 56 h after out‐of‐hospital death. One hundred forty‐nine tumour samples were snap‐frozen, of which 17 (4–7 per patient) were selected for whole‐genome sequencing. Phylogenetic reconstruction was performed using CONIPHER. Autopsy revealed multiple metastatic sites not visible on antemortem or postmortem imaging. *MYB‐NFIB* gene fusions were present across all tumour sites from the two AdCC patients, while a *LIFR‐PLAG1* fusion was detected in all samples from the MECA patient. All three cases showed extensive genetic tumour heterogeneity and branched phylogenies, suggestive of parallel evolution. Histologic growth patterns were consistent across metastatic sites. Overall, this study demonstrated marked genetic heterogeneity and branched evolution in these AdCC and MECA patients.

## Graphical abstract

Tumour heterogeneity and clonal evolution of metastatic salivary gland cancer were evaluated in two patients with adenoid carcinoma and one patient with myoepithelial carcinoma. Radiology‐guided autopsy enabled multi‐region sampling (total samples *n* = 149), followed by whole‐genome sequencing and phylogenetic reconstruction (17 tumour samples, 4–7 per patient). In these patients, extensive intermetastatic genetic heterogeneity and branched evolution were observed.

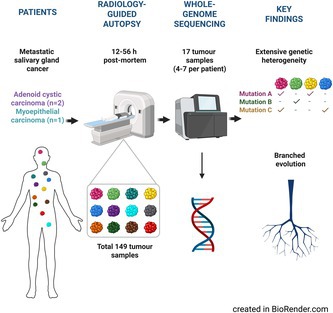

**Abbreviations**
AdCC: adenoid cystic carcinoma
CCF: cancer cell fraction
CONIPHER: correcting noise in phylogenetic evaluation and reconstruction
CT: computed tomography
MECA: myoepithelial carcinoma
R/M: recurrent and/or metastatic
SNV: single nucleotide variant
SGC: salivary gland cancer
WGS: whole‐genome sequencing

## 1 Introduction

Cancer clonal evolution—that is the process by which (epi)genetic alterations create diversity—is highly variable across cancer types and within single tumours [1, 2]. Inherent to these evolutionary dynamics is the introduction of genetic tumour heterogeneity, both within primary tumours and during the metastatic process [3, 4, 5]. Inversely, genetic heterogeneity can be leveraged to infer the temporal order of mutation events and can thereby resolve the evolutionary history of a cancer [3]. Metastatic evolution is generally described by two main models. In the linear model, tumour clones arise sequentially and dominate over time, with later dissemination from the primary tumour. In the parallel model, tumour cells disseminate earlier from the primary tumour, with metastases evolving independently and concurrently with the primary tumour [2, 5]. Alternative seeding processes can also contribute to metastatic diversity, including polyclonal seeding, primary tumour to metastasis reseeding and metastasis to metastasis reseeding [5].

These concepts of genetic tumour heterogeneity and clonal evolution have primarily been studied in common cancer types, but remain largely unexplored in salivary gland cancer (SGC) [6, 7]. Understanding genetic heterogeneity and clonal evolution of SGC is pivotal for therapy development and to guide treatment decisions, as tumour heterogeneity can drive resistance to (targeted) anticancer therapies, enabling resistant subclones to expand and become dominant [8]. This study focuses on two intercalated duct‐derived subtypes of SGC, adenoid cystic carcinoma (AdCC) and myoepithelial carcinoma (MECA) [9, 10]. AdCC and MECA share high rates of recurrent and/or metastatic (R/M) disease (55% and 42%, respectively) and a lack of systemic therapeutic options [11, 12].

AdCC typically arises from the major and minor salivary glands, but can originate in seromucinous glands throughout the body [13]. Recent proteogenomic analysis revealed that two molecular AdCC subtypes can be distinguished, with distinct phenotypes. AdCC‐I (37% of cases) has a more aggressive disease course (median overall survival 3.4 years), tends to metastasise to liver and bones, and is enriched with solid histology, upregulation of *MYC* signalling and *NOTCH*‐activating mutations. Conversely, AdCC‐II (63% of cases) follows a more indolent disease course (median overall survival 23.2 years), and is associated with non‐solid (tubular/cribriform) histology and upregulation of *TP63* and receptor tyrosine kinases. *TP63* and *MYC* gene expression alone were found to be sufficient to reliably classify AdCC tumours into these molecular subtypes [14]. In both molecular subtypes, gene fusions involving *MYB/MYBL1* and *NFIB* are common oncogenic drivers, being present in 72–88% of cases [15, 16, 17]. The efficacy of currently available palliative systemic treatments for AdCC is limited, with objective response rates of 15–25% for platinum‐based chemotherapy regimens and a pooled objective response rate of 6% for multi‐kinase inhibitors [18, 19].

Salivary MECA most commonly originates in the major salivary glands. It develops either *de novo* or from a pre‐existing pleomorphic adenoma, with a ratio of approximately 50 : 50 [12, 20, 21]. MECA mostly recurs locally, while fewer patients develop distant metastases [22]. Genetically, MECA is characterised by frequent fusions (70%), most commonly involving *PLAG1* [21]. The median overall survival following diagnosis is approximately 11 years [22]. Due to the rarity of the disease, the use of palliative systemic therapies has only been reported in small case series [23].

The limited efficacy of current palliative systemic therapies for these cancers underscores the need to analyse genetic tumour heterogeneity and related evolutionary dynamics, which may underlie resistance mechanisms. However, most current genomic studies focus on single samples, which restrict insights into genetic heterogeneity and clonal evolution [24]. Therefore, it is essential to complement these studies with multi‐region sampling. Autopsy provides a unique opportunity to collect large amounts of tissue from multiple tumour lesions, which is not otherwise possible in living patients [25].

In this study, we present an in‐depth analysis of the spatial genomic tumour landscape and evolutionary trajectories of two patients with end‐stage metastatic AdCC and one patient with end‐stage metastatic MECA. We use deep whole‐genome sequencing (WGS) on multiple spatially separated metastases, obtained through research autopsy. This study aimed to provide a deeper understanding of the spatial architecture of intrapatient tumour heterogeneity and the clonal evolution of AdCC and MECA.

## 2 Materials and methods

### 2.1 Patients

Four patients with metastatic SGC treated at the outpatient clinic of the Department of Medical Oncology of Radboud university medical centre, an SGC expertise centre in The Netherlands, participated in a research autopsy programme between October 2019 and January 2021. One patient with salivary duct carcinoma was excluded from later analyses in this study because tumour biopsies did not pass quality control due to extensive tumour necrosis caused by COVID‐19 related logistic constraints during autopsy. The study was approved by the institutional review board (Commissie Mensgebonden Onderzoek Radboudumc, file number 2019‐5089), and written informed consent was obtained from all participating patients. The study was conducted according to the standards set by the Declaration of Helsinki.

### 2.2 Transportation and body refrigeration

All patients died outside the hospital. Following declaration of death (by general practitioners as custom in The Netherlands, in accordance with all legal obligations), their bodies were immediately transported to Radboud university medical centre. Refrigeration to 2–5 °C commenced upon arrival and was initiated within 6 h after death.

### 2.3 Postmortem imaging

Postmortem imaging included computed tomography (CT) scanning of the neck, thorax and abdomen, and magnetic resonance imaging of the head, neck and brain. If technically feasible (i.e. depending on the degree of rigour mortis) and if no recent pulmonary antemortem imaging was available, intubation and inflation of the lungs took place during CT scanning to enhance visualisation of pulmonary metastases [26].

### 2.4 Autopsy and tumour sampling

Autopsy was commenced immediately after imaging. All internal organs were examined for tumour presence. Bones were selectively sampled where ante‐ or postmortem imaging indicated bone metastases. Brain autopsy was performed in two patients (patient A and B) who provided additional consent for this procedure. Representative tumour samples were collected from each affected organ. For pulmonary metastases, every invaded lobe was representatively sampled. Several large metastases were sampled from both the core and the rim. The primary tumour or a locoregional recurrence, if present, was also sampled. Healthy skin was sampled as a germline control. Samples were snap‐frozen in Tissue‐Tek OCT compound (Sakura) at −80 °C and formalin‐fixed and paraffin‐embedded.

### 2.5 Sample selection, DNA isolation, library preparation and WGS

Tumour purity was estimated on haematoxylin and eosin slides and most viable areas with highest tumour purity were annotated. For samples with sufficient viable tumour, punch biopsies of 2 mm were taken from the annotated areas for subsequent DNA isolation. DNA isolation, library preparation and WGS were performed at Hartwig Medical Foundation (Amsterdam, The Netherlands) according to previously published protocols [27]. A total of 20 tumour samples and healthy control samples were whole‐genome sequenced (Illumina Novaseq S4 2 × 150 bp V1.5) to a median coverage of 60× for tumour samples and 30× for healthy control samples. Selection of samples for WGS was based on the optimal relation between tumour purity (all > 25% on haematoxylin and eosin slides), concentration of prepared libraries and spatial distribution across different tumour locations in each patient. Prioritisation was given to metastases in different organs, rather than different metastases within one organ.

### 2.6 WGS analysis

WGS data were processed using Oncoanalyser (revision 0.4.1), a Nextflow [28] implementation of the Hartwig Medical Foundation pipeline. Oncoanalyser consists of 16 modules for the analysis of DNA sequencing data (see Table S1 for an overview). All sequencing reads were aligned to the GRCh38 reference genome. Somatic variant calling was performed using sage version 3.4, which applies tiered sensitivity based on the likelihood of pathogenicity. A predefined hotspot panel of 10 000 known variants was filtered at the highest sensitivity, followed by intermediate sensitivity for exonic and splice‐site regions for the canonical transcripts of a panel of cancer‐related genes. Genome‐wide filtering was performed more stringently, with distinct thresholds for high‐confidence and low‐confidence regions to ensure a low false positive rate outside targeted regions. Tumour heterogeneity of somatic mutations was assessed for genes listed in the Hartwig Medical Foundation driver mutation catalogue [27], and genes previously associated with the biology of AdCC [7, 15, 24] and MECA [21] (Table S2).

### 2.7 Phylogenetic reconstruction

The computational tool CONIPHER (Correcting Noise In Phylogenetic Evaluation and Reconstruction), which was integrated into a Nextflow based pipeline, was used for cancer cell fraction (CCF) based clustering and phylogenetic reconstruction [29]. As input for the Nextflow integration of CONIPHER, the somatic VCF files, tumour purity and tumour ploidy data generated by the Purple module from Oncoanalyser were used. Sex chromosomes were not included in the analysis. The clustering module was executed in two stages. In the first stage, the CCF was determined for each variant using a modified version of the Bayesian cluster model, pyclone [30]. Beta‐binomial emission densities were utilised to calculate the CCF per variant. Flexible prior probability estimation was then performed to correct for allelic prevalence in single nucleotide variants (SNVs) that have undergone copy number alteration events [30]. In the second stage, preclustering was performed by categorising variants based on their presence or absence across all tumour samples. These preclusters were further divided into subclusters using a Dirichlet distribution of the variant CCFs [31]. The resulting clusters were then used as input for the tree building module. Confidence intervals for each cluster were calculated from the CCF distribution of its constituent variants. An ancestral graph was constructed by assessing whether clusters could be nested within other clusters by performing two one‐sided Wilcoxon tests per cluster pair [29]. This ancestral graph was subsequently pruned to create a phylogenetic tree structure and clusters containing ≤ 5 SNVs were removed. The resulting tree was evaluated for cycles in the tree and for the presence of cluster CCFs at any particular tree level exceeding 10%. If any of these issues were detected, clusters were iteratively removed from the tree in ascending order of mutation count. The resulting tree structure was re‐tested for issues after each removal. This procedure was continued until a tree free of issue clusters was obtained, which was classified as the default tree. To explore alternative tree configurations, permutations of the initial tree were generated and ranked by sum condition error and highest edge probability [29]. The highest‐ranked tree, considered the most probable phylogenetic reconstruction, is presented.

### 2.8 RNA isolation and real‐time qPCR

RNA was extracted from five 20 μm tissue sections using TRIzol reagent (Thermo Fisher Scientific, Carlsbad, CA, USA) following the manufacturer's protocol (Thermo Fisher Scientific). After DNase I treatment, 2 μg of RNA was reverse transcribed into cDNA using SuperScript II Reverse Transcriptase following the manufacturer's instructions (Thermo Fisher Scientific). RNA integrity number (RIN) values were determined on a 2100 Bioanalyzer (Agilent Technologies, Santa Clara, CA, USA). Gene expression levels were determined in duplicates by SYBR Green qPCR using a LightCycler 480 (Roche, Basel, Switzerland). Gene expression levels of *TP63* and *MYC* were calculated using the 2^−ΔCt^ method with *GADPH* as the internal control. The primer sequences were as follows: *GAPDH* forward: 5′‐TCAAGGCTGAGAACGGGAA‐3′, reverse: 5′‐TGGACTCCACGACGTACTCA‐3′. *TP63* forward: 5′‐GTTTCGACGTGTCCTTCCAG‐3′, reverse: 5′‐TCTGGATGGGGCATGTCTTT‐3′. *MYC* forward: 5′‐TTCGGGTAGTGGAAAACCAG‐3′, reverse: 5′‐CACCGAGTCGTAGTCGAGGT‐3′.

### 2.9 Immunohistochemistry

Formalin‐fixed, paraffin‐embedded tissue blocks were sectioned at 4 μm thickness. Immunohistochemical staining was performed using fully automated protocols on a Dako Omnis autostainer (Agilent Technologies). For p63 staining, the DAK‐p63 mouse monoclonal antibody (Agilent Technologies) was used. For MYC staining, the ZR355 rabbit monoclonal antibody (Zeta Corporation, Tucson, AZ, USA) was applied. Both primary antibodies were diluted 1 : 50. Bound antibody was visualised using diaminobenzidine.

## 3 Results

### 3.1 Case histories, postmortem imaging and autopsy

**Fig. 1:**
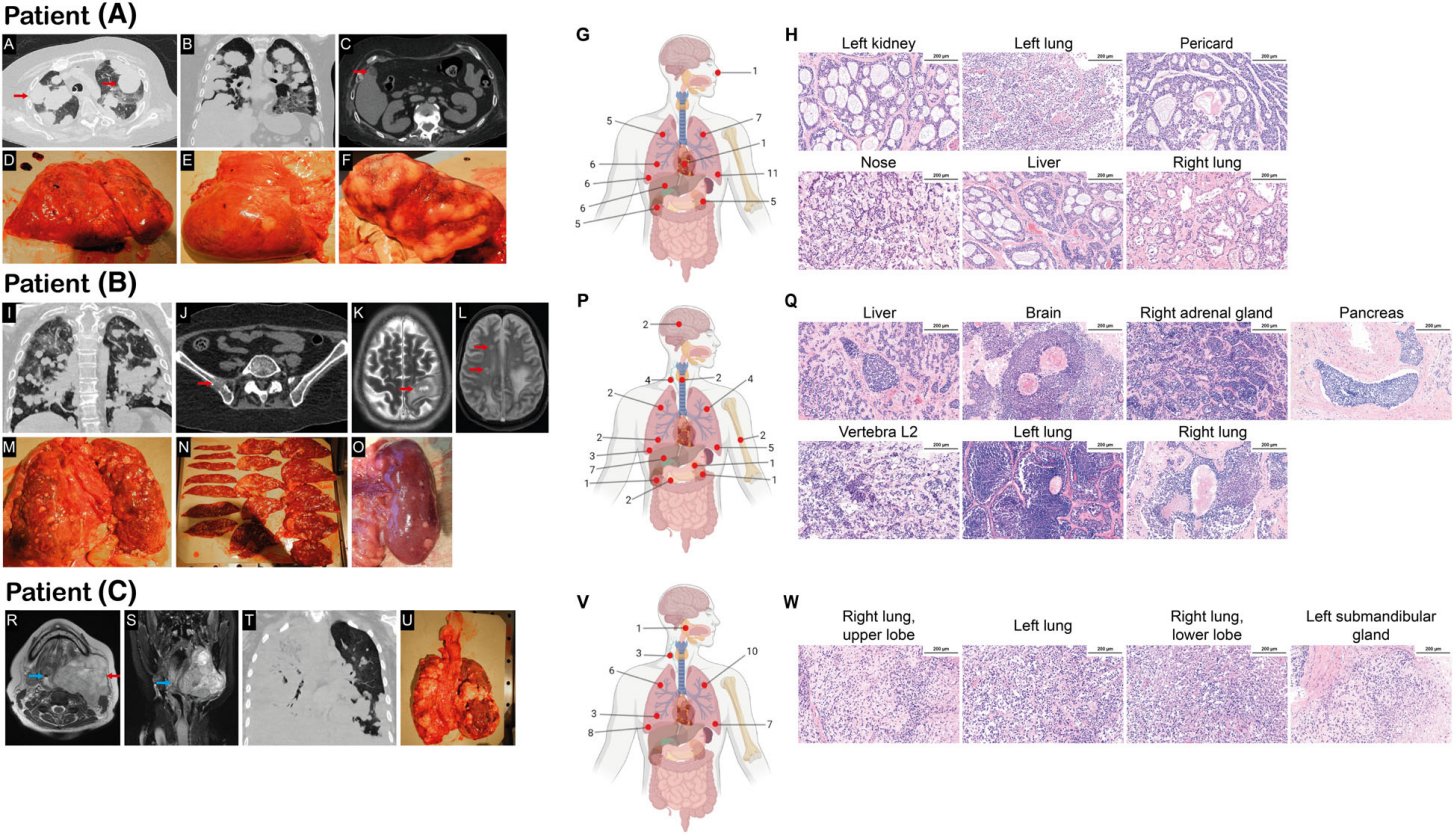
Postmortem radiological images, autopsy images, sampled tumour locations and histopathology across tumour sites. Patient A (adenoid cystic carcinoma): (A and B) Ventilated computed tomography (CT) scan of the lungs, displaying multiple large confluent metastases (arrows), transverse (A) and coronal (B) planes. (C) Transverse abdominal CT scan, revealing a metastasis in Segment 4 of the liver (arrow). (D) The left lung at autopsy, with multiple metastases. (E) The liver at autopsy, confirming the metastasis in Segment 4 of the liver. (F) The kidney at autopsy, showing numerous large metastases. (G) Overview of sampled tumour locations in patient A. Numbers indicate the number of samples containing sufficient non‐necrotic tumour cells per tumour location. (H) Representative haematoxylin and eosin (H&E) stainings of tumour samples from patient A at 20× magnification. The scale bar represents 200 μm. Patient B (adenoid cystic carcinoma): (I) Coronal CT scan of the lungs, showing extensive metastases in both lungs. (J) Transverse CT scan of the pelvis, indicating a metastasis in the right iliac bone (arrow). (K) T2‐weighted transverse magnetic resonance imaging (MRI) scan of the brain, showing a metastasis with oedema in the left parietal lobe. (L) T1‐weighted transverse MRI scan, revealing multiple smaller intracranial metastases. (M) The lungs at autopsy, confirming extensive metastases. (N) Serial sectioning of the liver, showing widespread metastases. (O) The kidney at autopsy, revealing multiple small metastases undetected on the CT scan. (P) Overview of sampled tumour locations in patient B. Numbers indicate the number of samples containing sufficient non‐necrotic tumour cells per tumour location. (Q) Representative H&E stainings of tumour samples from patient B at 20x magnification. The scale bar represents 200 μm. Patient C (myoepithelial carcinoma): (R) T2‐weighted transverse MRI scan, showing a large myoepithelial carcinoma originating from the left submandibular gland (red arrow), with compression on the trachea (blue arrow). (S) T1‐weighted fat‐saturated coronal MRI scan of the submandibular gland tumour. (T) Coronal CT scan illustrating extensive pulmonary metastases in both lungs and close to absent air‐containing parenchyma in the right lung. (U) Autopsy image of the left submandibular gland tumour and extensive lung metastases. (V) Overview of sampled tumour locations in patient C. Numbers indicate the number of samples containing sufficient non‐necrotic tumour cells per tumour location. (W) Representative H&E stainings of tumour samples from patient C at 20× magnification. The scale bar represents 200 μm.

Patient A, a 57‐year‐old female with a history of autoimmune hepatitis with primary biliary cirrhosis overlap, underwent a superficial parotidectomy after evaluation for a lump in the left neck area. Pathology revealed a pT2N0 AdCC with extensive perineural invasion and positive tumour margins. Postoperative radiotherapy (66 Gy) was administered. Approximately 1 year after completing postoperative radiotherapy, CT scanning revealed asymptomatic pulmonary and pleural metastases. Over the subsequent 6 years, these metastases gradually progressed, causing increasing dyspnoea and pain. Bilateral kidney metastases were identified on CT imaging 4 years after primary tumour resection, and a locoregional metastasis in the nasal vestibule was identified 5 years after resection. The patient opted out of palliative chemotherapy, and no actionable targets were identified with next‐generation sequencing of the primary tumour. Palliative radiotherapy (12 Gy) was administered to alleviate pain from pulmonary metastases invading the pleura. Eventually, 7 years after the primary diagnosis, the patient passed away due to respiratory failure caused by progression of the pulmonary metastases. Ventilated postmortem CT scanning confirmed extensive disease progression in both lungs, the nasal vestibule and both kidneys (Fig. 1A–C). Also, a liver metastasis in Segment 4 was detected, new compared with the most recent CT scan 12 months antemortem. Autopsy confirmed the findings of postmortem imaging and revealed several additional tumour lesions in the liver, as well as metastases in the pericardium and spleen (Fig. 1D–F), which were not visible on either the postmortem CT scan or the most recent antemortem CT scan 12 months prior to death.

Patient B, a 53‐year‐old female with a history of hypertension, underwent a combined mandibulectomy and neck dissection operation (Commando procedure) after evaluation for a tumour originating in the left submandibular gland. Pathology revealed a pT4N0 AdCC with a solid growth pattern, extensive perineural growth and positive tumour margins. Postoperative radiotherapy (70 Gy) was administered. Approximately 1 year after surgery, positron emission tomography/CT scanning revealed metastatic spread to the lungs, hilar and mediastinal lymph nodes, and vertebra L2. Next‐generation sequencing of the primary tumour identified no actionable targets. Three months after the diagnosis of metastatic disease, the patient experienced an epileptic seizure caused by multiple (> 20) brain metastases. CT scanning also showed further metastatic spread to the thyroid, the liver and multiple bones. Whole‐brain radiotherapy (20 Gy) and stereotactic radiotherapy for the two largest brain metastases (10 Gy) were administered. Also, palliative radiotherapy (8 Gy) was administered for a bone metastasis in the pelvis, and palliative systemic therapy with vinorelbine was initiated. Despite these interventions, rapid cognitive decline and disease progression manifested, leading to death 8 months after the diagnosis of metastatic disease. Postmortem imaging confirmed the evident disease progression in the lungs, mediastinal lymph nodes, thyroid, liver, skeleton and brain (Fig. 1I–L). Autopsy confirmed the findings of postmortem imaging and revealed additional metastases in both kidneys, the pancreas (head and tail), the right adrenal gland and a mesenteric lymph node (Fig. 1M–O), which were not detected by either the postmortem CT scan or the most recent antemortem CT scan 5 months prior to death.

Patient C, a female complaining of a lump at the mandibular angle, was diagnosed at the age of 48 years with MECA ex pleomorphic adenoma of the left submandibular gland with pulmonary and pleural metastases. Next‐generation sequencing of a pulmonary metastasis revealed a *LIFR‐PLAG1* fusion and loss of *CDKN2A*, upon which treatment with the CDK4/6 inhibitor ribociclib was initiated in a basket trial [32]. Five weeks after treatment initiation, the patient suffered from evident progression of the pulmonary and pleural metastases. Six months after the diagnosis, the patient died due to respiratory failure caused by the metastases along with pleural effusion. Postmortem imaging confirmed the extensive progression of the pulmonary and pleural metastases, along with substantial progression of the primary tumour, with a final diameter of 75 mm (Fig. 1R–T). These findings were confirmed during autopsy, with no evidence of extrapulmonary metastases (Fig. 1U).

### 3.2 Tumour sampling, library preparations and WGS

Autopsy was initiated between 12 and 56 h after death. During autopsy, a total of 149 samples of suspected tumour lesions were taken (58, 44 and 47 for patient A, B and C, respectively), as were healthy control samples for each patient (abdominal skin). In 129 out of 149 suspected tumour samples, sufficient non‐necrotic tumour was present to proceed to DNA isolation and library preparation (Fig. 1G,P,V). The yield after library preparations of all 129 tumour samples was sufficient for WGS, with an average library concentration of 13.51 nm. After selection, 17 tumour samples were whole‐genome sequenced, along with the three healthy control samples. The average WGS yield was 248 Mbase/sample (range: 212–323 Mbase).

### 3.3 Histological tumour heterogeneity

Histological evaluation demonstrated high intrapatient concordance alongside substantial inter‐patient variability, including a clear distinction between the two AdCC cases (Fig. 1H,Q,W). Tumour samples from patient A uniformly displayed a low‐grade mixed cribriform‐tubular architecture, whereas tumours from patient B consistently exhibited solid growth with high‐grade transformation with atypia, a relatively high mitotic rate and necrosis. All lesions from patient C displayed uniform morphological characteristics of MECA.

### 3.4 Heterogeneity in TP63 and MYC expression

**Fig. 2:**
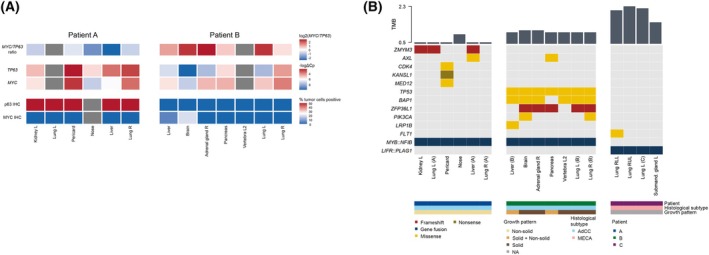
Expression of *TP63* and *MYC* and genetic alterations across tumour sites. (A) *TP63* and *MYC* expression in adenoid cystic carcinoma across tumour sites. Heatmaps show data from patient A and patient B (both adenoid cystic carcinoma). The upper three rows illustrate mRNA expression levels: the upper row displays the log_2_ ratio of *MYC*/*TP63* expression. The second and third rows show *TP63* and *MYC* expression levels (−logΔC*p*), respectively. The fourth and fifth rows display protein expression of p63 and MYC determined by immunohistochemistry (IHC), reported as the percentage of tumour cells positive. Grey boxes indicate samples with insufficient quality for analysis. (B) Genetic alterations across tumour sites in patient A (adenoid cystic carcinoma), patient B (adenoid cystic carcinoma) and patient C (myoepithelial carcinoma). Overview of exonic mutations, fusions and tumour mutational burden (TMB) across all sampled tumour lesions. AdCC, adenoid cystic carcinoma; L, left; NA, not applicable; R, right; RLL, right lower lobe; MECA, myoepithelial carcinoma; RUL, right upper lobe.

To identify molecular subtypes of AdCC, *TP63* and *MYC* gene expression and p63 and MYC immunohistochemical staining were evaluated in the tumour samples from both AdCC patients. The median RIN was 2.3 (range 2.0–2.5). Despite low RIN values, RNA quality was sufficient for gene expression analysis (*GAPDH* C*p*‐value < 32.0) in 11 out of 13 samples. Log_2_(*MYC*/*TP63*) ratios were consistently higher in patient B (median 1.92, range 0.50–2.78) than in patient A (median −0.69, range −2.56 to −0.31) (Fig. 2A; raw data provided in Table S3). Also, p63 immunohistochemical staining was consistently higher in patient A than in patient B. In patient A, 50% of the tumour cells were positive in all evaluable samples. In patient B, there was 0% positivity in all samples. MYC staining was low in both patients (representative images of the immunohistochemical staining provided in Fig. S2). Together with the histological growth patterns and clinical course, these results support classification of patient A as AdCC‐II and patient B as AdCC‐I, with high intrapatient concordance in *TP63* and *MYC* gene expression and p63 and MYC immunohistochemical staining.

### 3.5 Genetic tumour heterogeneity

**Fig. 3:**
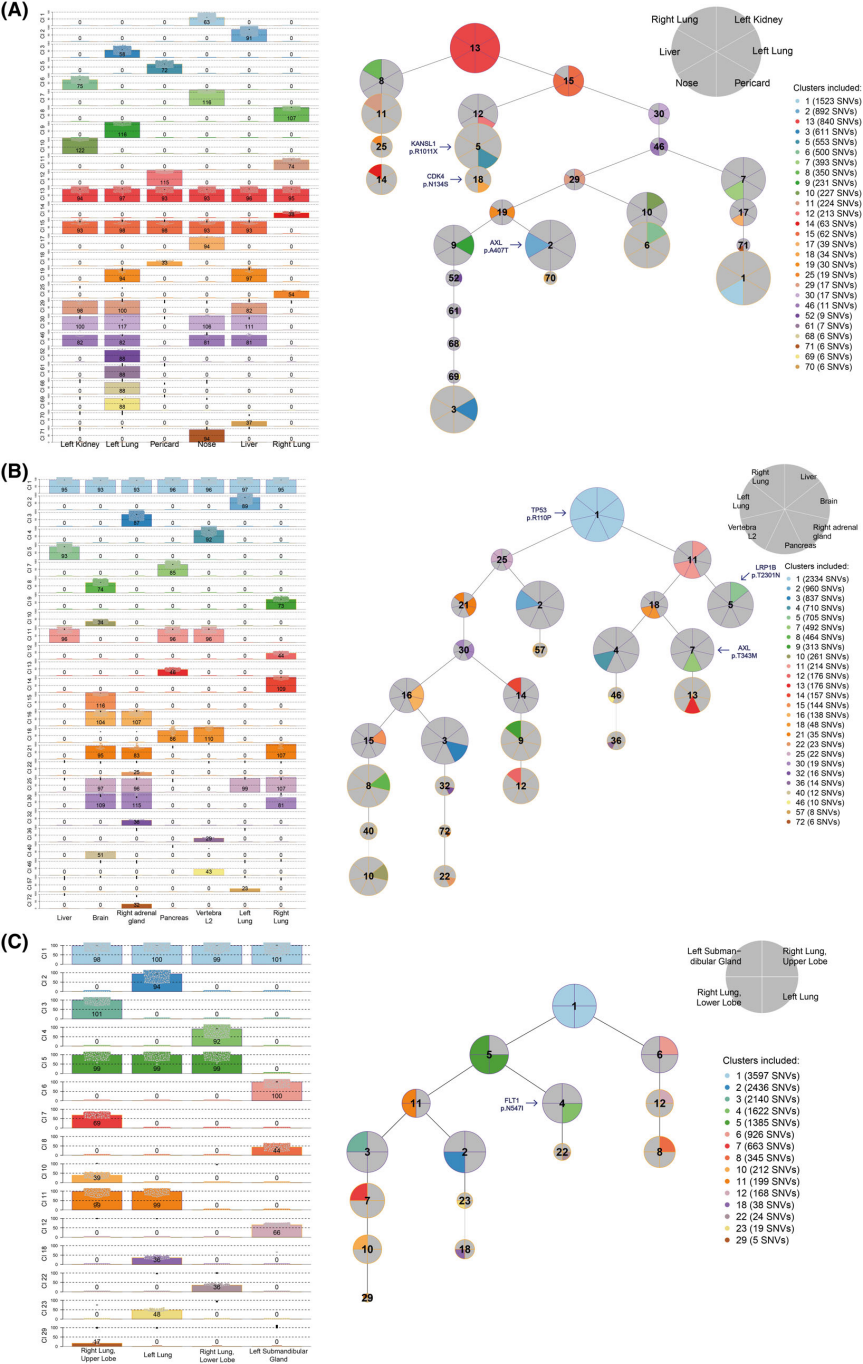
Phylogenetic trees and cancer cell fraction (CCF) bar plots. Panels A, B and C represent patients A (adenoid cystic carcinoma), B (adenoid cystic carcinoma) and C (myoepithelial carcinoma), respectively. Each node represents a variant cluster, with node size proportional to the number of variants in that cluster. Cluster numbers (shown within the nodes) are ordered by variant count. The number of variants within a cluster is specified in the ‘clusters included’ legend. Each node is divided into segments that correspond to tumour sample sites shown in the pie chart legend. Nodes with outer and inner lines in purple denote clonal clusters (CCF ≥ 80%), whereas nodes with orange lines denote subclonal clusters (CCF < 80%). Each cluster is assigned a unique colour matching the bars in the CCF bar plots. The bars represent the CCF of variants for each tumour sample. Exonic variants are displayed with arrows pointing to their associated clusters. Not all exonic variants are displayed due to their presence in excluded clusters (*BAP1* and *PIK3CA* in patient B), their location on sex chromosomes (*MED12* and *ZMYM3* in patient A), or because variants other than single nucleotide variants were not included in the phylogenetic reconstruction (frameshift deletion of *ZMYM3* in patient A and frameshift insertion of *ZFP36L1* in patient B). SNV, single nucleotide variant.

Exonic genetic alterations and gene fusions across the tumour samples of all three patients are displayed in Fig. 2B, and an overview of associated pathways and potential actionability of the exonic mutations is provided in Table S4. In patient A, a *MYB‐NFIB* gene fusion was present in all samples. A *ZMYM3* frameshift deletion was identified in three lesions, while mutations in *KANSL1*, *AXL*, *CDK4* and *MED12* were restricted to single tumour sites. The predominance of alterations confined to single lesions suggests a high degree of genetic heterogeneity. Consistent with these observations, the phylogenetic tree of patient A reveals a highly branched structure (Fig. 3A). Only a few mutation clusters are shared across multiple tumour sites, while the majority—including several of the largest—are only present in one tumour lesion. The number of SNVs in the inferred primary tumour is relatively low. Together, these features indicate extensive genetic tumour heterogeneity and strengthen the case for parallel evolution.

Patient B also harboured a *MYB‐NFIB* gene fusion in all tumour sites, and, unlike patient A, carried multiple non‐fusion variants that were shared among most tumours. A *TP53* mutation was present in all tumours, a *BAP1* mutation was absent only in the pancreatic lesion, and a *ZFP36L1* frameshift insertion was detected in all tumours except the liver and vertebra L2 metastases. The consistent presence of these alterations across tumour sites likely reflects early clonal driver events. Further, a *PIK3CA* mutation was identified in two tumour lesions, and *AXL* and *LRP1B* mutations were restricted to single tumour sites. The phylogenetic tree of patient B displays a highly branched structure with two major genomically distinct branches emerging from the primary tumour (Fig. 3B). It predominantly consists of clusters that are exclusive to individual tumour samples, but also contains clusters that are shared among metastatic sites, unlike patient A. Besides this, the inferred primary tumour in patient B contains a higher number of SNV. Overall, this tree also suggests parallel evolution, but with a smaller degree of genetic tumour heterogeneity and a later onset of tumour dissemination compared with patient A.

Patient C carried a *LIFR‐PLAG1* fusion across all tumour samples. Furthermore, only a single exonic mutation in the *FLT1* gene, encoding the VEGFR1 protein, was detected exclusively in the right lower lobe lung lesion. Despite the paucity of exonic variants, patient C exhibited the highest tumour mutational burden among the three patients with 1.5–2.3 mutations per Mb, primarily resulting from noncoding mutations. The phylogenetic tree of patient C illustrates a relatively simple evolutionary structure (Fig. 3C). The primary left submandibular gland tumour branches off earliest, reflecting high genetic divergence. The three lung lesions cluster together on the opposite side of the tree, sharing the large cluster 5, which reflects genetic similarity and suggests a common metastatic progenitor. Most clusters are exclusive to individual tumour samples, suggesting parallel evolution. However, similar to patient B, the relatively high number of SNVs in the inferred primary tumour suggests smaller genetic tumour heterogeneity and a later stage of dissemination compared with patient A.

For all three patients, exonic variants represented only a minority of all detected somatic alterations. An overview of the number of mutations per genomic site is provided in Fig. S1.

### 3.6 Copy number heterogeneity

**Fig. 4:**
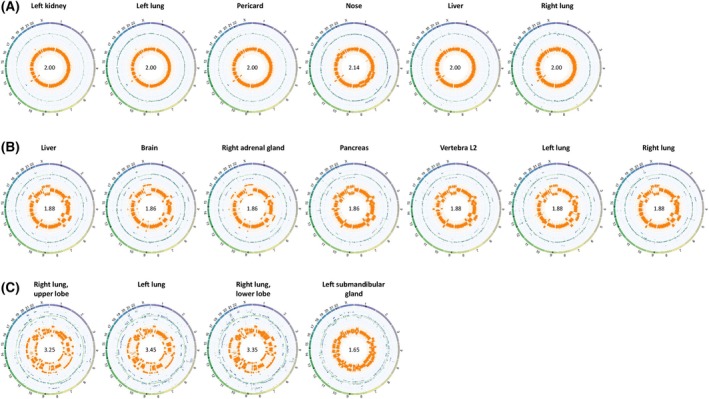
Copy number profiles across tumour sites. The copy number plots provide an overview of the copy numbers in patient A (adenoid cystic carcinoma), patient B (adenoid cystic carcinoma) and patient C (myoepithelial carcinoma). The outermost ring represents the 22 chromosomes and the X chromosome. The middle ring displays copy numbers in the healthy (green) and the tumour (blue) samples, with an axis ranging from 0 (inside axis) to 4 (outside axis). The innermost ring shows the B‐allele frequency in the tumour samples, representing the proportion of sequencing reads supporting the alternate allele relative to the reference genome, with values ranging from 0 (reference homozygous, inner axis) through 0.5 (alternate heterozygous, middle axis) to 1 (alternate homozygous, outer axis). The number in the centre of the rings represents overall ploidy.

Copy number profiles per tumour sample were also determined and are shown in Fig. 4. In patient A, overall ploidy was 2.0 for most tumour sites, except for the nasal lesion, where the ploidy increased to 2.14 due to amplifications on Chromosomes 5, 7 and 18 (Fig. 4A). In patient B, ploidy was stable between 1.86 and 1.88 across all tumour sites. Recurrent alterations included complete copy loss in Chromosomes 17 and X, and partial copy loss in Chromosomes 3, 5, 6, 9, 11 and 22 (Fig. 4B). In patient C, the primary tumour arising from the left submandibular gland exhibited a lower ploidy of 1.62, while all metastatic samples displayed ploidy levels between 3.25 and 3.45 due to a whole‐genome duplication (Fig. 4C).

## 4 Discussion

This study represents, to our knowledge, the first research autopsy programme in metastatic SGC, providing insights into its intrapatient tumour heterogeneity and clonal evolution. Focusing on the histological subtypes AdCC and MECA, our findings reveal a high degree of intermetastatic genetic heterogeneity within the studied patients, although copy number profiles and ploidy were more concordant across metastatic sites. Phylogenetic reconstruction suggested a parallel evolutionary trajectory characterised by branched evolution, which may reflect early dissemination.

The current literature on genetic tumour heterogeneity and clonal evolution in AdCC and MECA is limited, largely due to the rarity of these malignancies. Liu *et al*. performed phylogenetic reconstruction in eight AdCC patients using multi‐region sampling of the primary tumour but included only a small number of distant metastatic lesions (*n* = 1 in seven patients; *n* = 2 in one patient) [6]. In their study, substantial genetic tumour heterogeneity and branched evolution with parallel dissemination was observed in all cases. In another study, Ho *et al*., [7] performed multi‐region sequencing of the primary tumour and eight metastatic lung lesions in one AdCC patient and also found evidence for marked genetic heterogeneity and branching evolution. Our findings are consistent with the patterns described in these studies, also suggesting branched, parallel evolution. In MECA, prior insights into genetic tumour heterogeneity and clonal evolution are limited to a study of Dalin *et al*. [21]. In their study, genetic heterogeneity in MECA was suggested through the presence of at least one subclonal population in the majority of tumours, but its conclusions were drawn from single tumour samples per patient. Our study confirms substantial genetic heterogeneity in this MECA patient, driven by parallel clonal evolution.

The observed intrapatient genetic heterogeneity may play a role in drug resistance in AdCC and MECA. Several of the alterations identified – including *ZMYM3*, *MED12*, *TP53*, *BAP1*, *PIK3CA* and *LRP1B*—have previously been implicated in resistance to systemic anticancer therapies [33, 34, 35, 36, 37, 38]. As these alterations were frequently restricted to a subset of tumour regions within the same patient, the sensitivity for anticancer drugs may differ between tumour sites. Although the demonstrated genetic heterogeneity may generate hypotheses regarding its role in therapeutic resistance, this is an important area for future research and not a conclusion that can be drawn from the current study, given the limited treatment exposure, lack of temporal sampling and absence of functional validation. Moreover, the genetic heterogeneity highlights a major limitation of single‐region tumour biopsies: they risk underestimating the genomic complexity and may fail to capture clinically relevant resistance mechanisms. Liquid biopsy offers a promising strategy to address these challenges by enabling the detection of genetic spatial heterogeneity within individual patients and reducing sampling bias [39, 40]. Although still understudied in SGC, initial experiences with circulating tumour DNA have shown its potential for disease monitoring and the prediction of treatment response [41, 42].

Recent studies have established that molecular subtype classification of AdCC is a strong predictor of disease course [14, 24]. In our study, patient A displayed features characteristic of the AdCC‐II phenotype, including a long metastasis‐free interval, non‐solid histology and lower *MYC*/*TP63* gene expression ratios, whereas patient B exhibited the typical AdCC‐I phenotype with a short metastasis‐free interval, solid histology and higher *MYC*/*TP63* gene expression ratios. The highest degree of genetic heterogeneity was observed in the patient with the AdCC‐II phenotype, while the patient with the AdCC‐I phenotype harboured more clonal driver mutations that were shared across metastatic sites. Based on these two cases, it could be hypothesised that AdCC‐II exhibits more genetic heterogeneity than AdCC‐I, in line with the theory that intermetastatic heterogeneity tends to increase when the primary tumour grows slowly [1]. Furthermore, in both AdCC patients, growth patterns and *TP63* and *MYC* gene expression remained consistent across metastatic sites, suggesting that a single metastatic sample may be sufficient to determine the AdCC molecular subtype within an individual patient, while requiring validation in a larger patient cohort. The consistent *TP63* and *MYC* expression across metastatic sites supports treatment stratification based on *TP63* and *MYC* gene expression defined subtypes, which may be valuable in addition to therapeutic decision‐making based on genetic alterations. Such a subtype‐based approach is supported by a recent clinical trial demonstrating better clinical outcomes with axitinib and avelumab in AdCC‐II compared with AdCC‐I [43], while AdCC‐I specific targets are also being explored, such as B7‐H4 [44].

In our study, copy number profiles and ploidy were largely concordant across metastatic sites. An exception was the MECA patient, where a whole‐genome duplication was present in the three lung metastases, but not in the primary tumour. These findings align with a previous pan‐cancer study demonstrating that karyotypes tend to be conserved across metastatic lesions, while structural variants are more prevalent in metastatic tumours compared with primary tumours in the majority of cancer types [45].

Furthermore, this study shows that tumour tissue with sufficient DNA integrity for WGS and phylogenetic reconstruction can be obtained from patients who died outside the hospital, with a postmortem interval up to 56 h. In addition, RNA integrity was sufficient for RT‐qPCR analyses in 11 out of 13 samples. It has previously been shown that the impact of postmortem interval on nucleic acid integrity is less consistent in tumour tissue compared with normal tissue [46, 47]. Also in the Posthumous Evaluation of Advanced Cancer Environment (PEACE; NCT03004755) autopsy programme [48], a shorter postmortem interval correlated only weakly with higher DNA integrity number. In the same study, RNA sequencing was successfully performed with a median postmortem interval of 52 h (range 23–144 h). Together, these findings support the feasibility of postmortem tissue collection for molecular analyses not only in controlled ‘rapid autopsy’ settings, but also in patients who die at home—provided that timely transport and refrigeration are ensured. Additionally, our protocol with annotation of the most viable and tumour‐rich areas prior to punch biopsies for library preparations may have contributed to the high success rate of library preparations.

Interestingly, autopsy of both AdCC patients revealed tumour sites that were not detected by either antemortem or postmortem radiological scans. Although the interval between antemortem scanning and autopsy and the absence of contrast enhancement in the postmortem scans may have contributed to these discrepancies, our findings suggest that metastases in less common sites, such as the pericardium, spleen, kidneys and pancreas, may be more prevalent than previously recognised, but remain frequently undetected by conventional imaging modalities. This aligns with observations from a breast cancer autopsy study, in which seven out of nine patients exhibited extensive tumour involvement of additional organs not visible on imaging [49], emphasising the limitations of radiological imaging in fully capturing metastatic regions.

Our study has several limitations. First, the small number of patients and the inclusion of only two out of 21 salivary gland cancer subtypes limits the generalisability of our findings. Larger cohorts are warranted to validate these results and enhance their representativeness. Also, autopsy studies in other salivary gland cancer subtypes are needed to generate insights into their tumour heterogeneity and evolutionary patterns. However, the rarity of the disease and the resource‐intensive nature of research autopsy programmes pose significant challenges. Second, although a total of 129 tumour samples with sufficient DNA yield were obtained, only 17 samples (4–7 per patient) were included in the phylogenetic analysis due to budgetary constraints. Third, only one sample per metastasis was sequenced. This approach may lead to underestimation of intralesional heterogeneity and could create an illusion of clonality, as subclonal diversity restricted to unsampled regions within the metastasis may remain undetected [3]. Fourth, the primary tumour was only available in the phylogenetic analysis of patient C, but not in the two AdCC patients. This reflects common clinical practice, as AdCC patients typically develop metachronous metastases, often years after primary tumour resection. Notably, CONIPHER enables reliable phylogenetic reconstruction without the primary tumour being required, which is one of the advantages of CONIPHER over other reconstruction models. Moreover, CONIPHER provides improved accuracy through the exclusion of biologically improbable clusters and correction for complex evolutionary events [29]. Finally, the averaging inherent to bulk DNA sequencing masks subclonal complexity and limits the resolution of phylogenetic resolution. Single‐cell DNA sequencing could overcome this limitation by preserving cellular heterogeneity, which enables more precise reconstruction of tumour evolutionary trajectories [50, 51, 52].

## 5 Conclusions

In conclusion, this study demonstrates spatial genetic heterogeneity in AdCC and MECA using a unique autopsy‐based WGS dataset. Future studies will be important to clarify the biological and clinical implications of this heterogeneity.

## Conflict of interest

The authors declare no conflict of interest.

## Author contributions

GL and CMLH conceived and designed the study and acquired funding. GL, NJR, CA, LO, PYL, WMK, ACHE‐G, JAS, SB, GWV and CMLH contributed to the methodology. GL, NJR, PYL, WMK, ACHE‐G and CMLH conducted the investigation. GL, NJR, CA, LO and SB curated the data. GL and NJR performed the formal analyses. NJR and CA prepared the visualisations. JAS, SB, GWV, ACHE‐G, CMLH, CA, LO, PYL and WMK supervised the project. GL and NJR wrote the original draft of the manuscript. CA, LO, PYL, WMK, ACHE‐G, JAS, SB, GWV and CMLH reviewed and edited the manuscript. All authors read and approved the final manuscript.

## Supporting information


**Table S1.** Oncoanalyser 0.4.1 tools and versions.
**Table S2.** List of genes previously associated with the biology of adenoid cystic carcinoma and myoepithelial carcinoma.
**Table S3.** Raw real‐time qPCR data, including duplicate and mean Cp values for *GAPDH*, *MYC* and *TP63*, with corresponding log2(*MYC*/*TP63*) expression ratios.
**Table S4.** Overview of detected exonic mutations, associated pathways and potential actionability.
**Fig. S1.** Overview of the number of alterations identified by whole‐genome sequencing per genomic site in patient A, patient B and patient C.
**Fig. S2.** Representative images of p63 and MYC immunohistochemical staining of patient A and patient B.

## Data Availability

The data that support the findings of this study are openly available in the European Genome Phenome Archive at https://www.ega‐archive.org/studies/EGAS50000001420.
