# Supplementary material for: Intrapatient tumour heterogeneity and clonal evolution in an autopsy study of metastatic salivary gland cancer
Source: Mol Oncol. 2026 Aug 6:10.1002/1878-0261.70315. Online ahead of print. doi: 10.1002/1878-0261.70315 (PMC13447987; doi:10.1002/1878-0261.70315)
Supplement: Supplementary file 1 [file MOL2-9999-0-s001.docx]

**Supplementary Material
*Molecular Oncology***

**Intrapatient tumour heterogeneity and clonal evolution in metastatic salivary gland cancer: an autopsy study**

**Authors names**
Gerben Lassche^#,1^, Niels J. van Ruitenbeek^#,1^, Charlotte Adang^2^, Luke O’Gorman^2^, Pui Yuen Lee^3^, Willemijn M. Klein^4^, Adriana C.H. van Engen-van Grunsven^3^, Jack A. Schalken^5^, Sander Bervoets^#,2^, Gerald W. Verhaegh^#,5^, Carla M.L. van Herpen^1^

^#^Contributed equally.

**Addresses**
^1^ Department of Medical Oncology, Radboud Institute for Medical Innovation, Radboud University Medical Center, Nijmegen, The Netherlands. ^2^ Radboud Technology Center for Bioinformatics, Radboud Institute for Molecular Life Sciences, Radboud University Medical Center, Nijmegen, The Netherlands.
^3^ Department of Pathology, Radboud Institute for Medical Innovation, Radboud University Medical Center, Nijmegen, The Netherlands.
^4^ Department of Medical Imaging, Radboud Institute for Medical Innovation, Radboud University Medical Center, Nijmegen, The Netherlands.
^5^ Department of Urology, Radboud Institute for Medical Innovation, Radboud University Medical Center, Nijmegen, The Netherlands.

**Corresponding author**:
Prof. dr. Carla van Herpen, Department of Medical Oncology, Radboud University Medical Center, P.O. Box 9101, 6500 HB Nijmegen, The Netherlands, Tel.: +31-24-3667251, e-mail address: [Carla.vanHerpen@radboudumc.nl](mailto:Carla.vanHerpen@radboudumc.nl)

**Supplementary tables**

**Supplementary table 1:** Oncoanalyser 0.4.1 tools and versions. Oncoanalyser uses modules that are a Nextflow integration of already existing Hartwig Medical Foundation tools and DNA alignment tools. Revision 0.4.1 of Oncoanalyser used the versions mentioned in this table for each of its tools.

| **Tool** | **Version** |
| --- | --- |
| Amber | 4.0 |
| Bamtools | 1.2.1 |
| BWA-mem2 | 2.2.1 |
| Chord | 2.3 |
| Cobalt | 1.16 |
| Cuppa | 1.8.1 |
| Gridss | 2.13.2 |
| Gripss | 2.4 |
| Lilac | 1.6 |
| Linx | 1.25 |
| Orange | 2.7.1 |
| Pave | 1.6 |
| Purple | 4.0.2 |
| Sage | 3.4 |
| Sambamba | 1.0.0 |
| Virusbreakend | 1.3 |

**Supplementary table 2:** List of genes previously associated with the biology of adenoid cystic carcinoma and myoepithelial carcinoma

| **Adenoid cystic carcinoma** | | **Myoepithelial carcinoma** |
| --- | --- | --- |
| *AKT1* | *MGA* | *ATM* |
| *ARID1A* | *MGEA5* | *ATR* |
| *ARID1B* | *MLL3/KMT2C* | *BRCA1* |
| *ARID4B* | *MLL3/KMT2D* | *CDKN2A* |
| *ARID5B* | *MUC16* | *COL2A1* |
| *ATM* | *MUC4* | *FAT1* |
| *BCOR* | *NAA16* | *FAT4* |
| *BRCA1* | *NOTCH1* | *FGFR1* |
| *CALM2* | *NOTCH2* | *FGFR2* |
| *CREBBP* | *NOTCH3* | *HRAS* |
| *CTNNB1* | *NOTCH4* | *KMT2C* |
| *EP300* | *PIK3CA* | *LIFR* |
| *ERCC2* | *PIK3R1* | *MAML2* |
| *FAT3* | *PTEN* | *MET* |
| *FAT4* | *PTPRG* | *MN1* |
| *FBXW7* | *PTPRH* | *NCOA1* |
| *FGF16* | *PTPRK* | *NOTCH1* |
| *FGFR2* | *pTERT** | *PCM1* |
| *FGFR4* | *RUNX1* | *PIK3CA* |
| *FOXO3* | *RYR2* | *SETD2* |
| *FOXP2* | *RYR3* | *SMARCA4* |
| *FTSJD1* | *SETD2* | *TRIP11* |
| *KDM6A* | *SMARCA1* |  |
| *KRAS* | *SMARCA2* |  |
| *LRP1B* | *SPEN* |  |
| *MAGI1* | *TLN1* |  |
| *MAGI2* | *TP53* |  |
| *MAP2K2* | *UHRF1* |  |

*, *TERT* gene promoter.

**Supplementary table 3:** Raw real-time qPCR data, including duplicate and mean Cp values for *GAPDH*, *MYC*, and *TP63*, with corresponding log2(*MYC*/*TP63*) expression ratios.

|  | **GAPDH Cp** | **GAPDH Cp, mean** | **MYC Cp** | **MYC Cp, mean** | **TP63 Cp** | **TP63 Cp, mean** | **Log2(MYC/TP63) ratio** |
| --- | --- | --- | --- | --- | --- | --- | --- |
|  |  |  |  |  |  |  |  |
| **Patient A** |  | | | | | | |
| Kidney L | 26,88 | 26,75 | 32,48 | 32,21 | 31,34 | 31,53 | -0,69 |
|  | 26,61 |  | 31,94 |  | 31,71 |  |  |
| Lung L | 36,00 | 36,19 | NA | NA | NA | NA | NA |
|  | 36,39 |  | NA |  | NA |  |  |
| Pericard | 29,47 | 29,82 | 33,10 | 32,39 | 31,99 | 32,08 | -0,31 |
|  | 30,16 |  | 31,68 |  | 32,17 |  |  |
| Nose | 28,96 | 28,99 | 33,91 | 36,08 | 34,64 | 34,31 | -1,78 |
|  | 29,01 |  | 34,70 |  | 33,94 |  |  |
| Liver | 27,70 | 27,64 | 33,93 | 33,70 | 31,15 | 31,13 | -2,56 |
|  | 27,58 |  | 33,46 |  | 31,11 |  |  |
| Lung R | 30,74 | 31,17 | 34,23 | 34,59 | 34,15 | 33,93 | -0,66 |
|  | 31,60 |  | 34,94 |  | 33,71 |  |  |
| **Patient B** |  | | | | | | |
| Liver | 27,96 | 28,07 | 33,45 | 33,53 | 34,48 | 34,82 | 1,29 |
|  | 28,18 |  | 33,60 |  | 35,15 |  |  |
| Brain | 27,24 | 27,29 | 34,20 | 34,37 | 36,73 | 36,93 | 2,56 |
|  | 27,33 |  | 34,53 |  | 37,12 |  |  |
| Adrenal gland R | 27,22 | 27,29 | 32,44 | 32,01 | 34,74 | 34,79 | 2,78 |
|  | 27,36 |  | 31,58 |  | 34,83 |  |  |
| Pancreas | 29,24 | 29,23 | 33,91 | 33,82 | 34,71 | 34,59 | 0,77 |
|  | 29,21 |  | 33,72 |  | 34,46 |  |  |
| Vertebra L2 | 32,68 | 33,36 | NA | NA | NA | NA | NA |
|  | 34,04 |  | NA |  | NA |  |  |
| Lung L | 26,75 | 26,66 | 31,74 | 31,66 | 34,57 | 34,23 | 2,57 |
|  | 26,56 |  | 31,57 |  | 33,88 |  |  |
| Lung R | 30,70 | 30,28 | 34,79 | 34,01 | 34,29 | 34,51 | 0,50 |
|  | 29,85 |  | 33,22 |  | 34,73 |  |  |

NA, not applicable.

| **Alteration** | **Associated pathways^a^** | **Potential actionability** |
| --- | --- | --- |
| **Patient A** | | |
| ZMYM3 p.R810fs | DNA damage repair, chromatin remodelling [1] | None identified |
| AXL p.A407T | Vascular endothelial growth factor pathway, receptor tyrosine kinases pathway (Reactome) | AXL-targeting antibody-drug conjugates (*e.g.*, mecbotamab vedotin) |
| CDK4 p.N134S | Cell cycle (Reactome) | CDK4/6 inhibitors (*e.g.*, palbocilib, ribociclib, abemaciclib) |
| KANSL1 p.R1011* | Chromatin organization (Reactome) | None identified |
| MED12 p.Q2120H | Epigenetic regulation of gene expression (Reactome) | None identified |
| **Patient B** | | |
| AXL p.T343M | Vascular endothelial growth factor pathway, receptor tyrosine kinases pathway (Reactome) | AXL-targeting antibody-drug conjugates (*e.g.*, mecbotamab vedotin) |
| TP53 p.R110P | Apoptosis, cell cycle, cellular senescence, DNA damage repair (Reactome) | None identified |
| BAP1 p.D663V | DNA damage repair (Reactome) | EZH2 inhibitors (*e.g.,* tazemetostat) [2] |
| ZFP36L1 p.A78fs | Cellular senescence (KEGG) Regulation of mRNA stability (Reactome) | None identified |
| PIK3CA p.E542K | PI3K/AKT/mTOR pathway (Reactome) | PI3Kα inhibitors (*e.g.*, alpelisib) |
| LRP1B p.T2301N | Tumour suppression gene [3] | None identified |
| **Patient C** | | |
| FLT1 p.N547I | Vascular endothelial growth factor pathway (Reactome) | None identified |

**Supplementary table 4:** Overview of detected exonic mutations, associated pathways, and potential actionability.

^a^ Selected pathways from Reactome and the Kyoto Encyclopedia of Genes and Genomes (KEGG). When no relevant pathways were identified in these databases, additional literature was searched.

**Supplementary figures**

**Supplementary figure 1:** Overview of the number of alterations identified by whole-genome sequencing per genomic site in patient A (adenoid cystic carcinoma), patient B (adenoid cystic carcinoma), and patient C (myoepithelial carcinoma). All alterations are included once, independent of the presence in multiple tumour samples.
ncRNA, non-coding RNA; UTR, untranslated region.

**Supplementary figure 2:** Representative images of p63 and MYC immunohistochemical staining of (A) patient A and (B) patient B. The scale bars represent 200 μm.

1.	Rodríguez-Sánchez, A., et al., *ZMYM3 Mutations Cooperate with NOTCH1 Alterations, Reduce Histone H4 Acetylation and Promote Apoptosis Evasion in Chronic Lymphocytic Leukemia.* Blood, 2023. **142**(Supplement 1): p. 1886-1886.

2.	Zauderer, M.G., et al., *EZH2 inhibitor tazemetostat in patients with relapsed or refractory, BAP1-inactivated malignant pleural mesothelioma: a multicentre, open-label, phase 2 study.* Lancet Oncol, 2022. **23**(6): p. 758-767.

3.	Cao, C.H., et al., *LRP1B mutation is associated with tumor HPV status and promotes poor disease outcomes with a higher mutation count in HPV-related cervical carcinoma and head & neck squamous cell carcinoma.* Int J Biol Sci, 2021. **17**(7): p. 1744-1756.

**References**
